# Supplementary material for: Direct evidence of acid-driven protein desolvation
Source: Proc Natl Acad Sci U S A. 2026 Mar 5;123(10):e2525949123. doi: 10.1073/pnas.2525949123 (PMC12974452; doi:10.1073/pnas.2525949123)
Supplement: Supplementary file 1 — Appendix 01 (PDF) [file pnas.2525949123.sapp.pdf]

PNAS

Supporting Information for

Direct evidence of acid-driven protein desolvation

Farzad Hamdi[#1,2], Ioannis Skalidis[#1,3], Inken Kaja Schwerin[#4], Jaydeep Belapure[1,5], Dmitry A. Semchonok[1,6], Fotis L. Kyrilis[1,7], Christian Tüting[1,2], Johannes Müller[8], Georg Künze[4,9,10], Panagiotis L. Kastritis[1,2,7,11]*.

[1]Department of Integrative Structural Biochemistry, Institute of Biochemistry and Biotechnology, Martin Luther University Halle-Wittenberg, Kurt-Mothes-Straße 3, 06120 Halle/Saale, Germany

[2]Interdisciplinary Research Center HALOmem, Charles Tanford Protein Center, Martin Luther University Halle-Wittenberg, Kurt-Mothes-Straße 3a, 06120 Halle/Saale, Germany.

[3]Structural Biochemistry, Bijvoet Centre for Biomolecular Research, Utrecht University, 3584 CG Utrecht, the Netherlands

[4]Institute for Drug Discovery, Leipzig University, Brüderstraße 34, 04103 Leipzig, Germany

[5]Navigo Proteins GmbH, 06120, Halle/Saale, Germany.

[6]Instituto de Tecnologia Química e Biológica António Xavier, Universidade Nova de Lisboa. Av. da República, 2780-157 Oeiras, Portugal.

[7]Institute of Chemical Biology, National Hellenic Research Foundation, 11635 Athens, Greece

[8]Institute of Pharmacy, Martin-Luther-University Halle-Wittenberg, Weinbergweg 22, D-06120 Halle (Saale), Germany.

[9]Interdisciplinary Center for Bioinformatics, Leipzig University, 04107 Leipzig, Germany

[10]Center for Scalable Data Analytics and Artificial Intelligence, Leipzig University, 04105 Leipzig, Germany

[11]Biozentrum, Martin Luther University Halle-Wittenberg, Weinbergweg 22, 06120 Halle/Saale, Germany.

*Panagiotis L. Kastritis, corresponding author.
[#]equally contributed
Email: panagiotis.kastritis@bct.uni-halle.de

This PDF file includes:

Figures S1 to S15
Tables S1 to S4

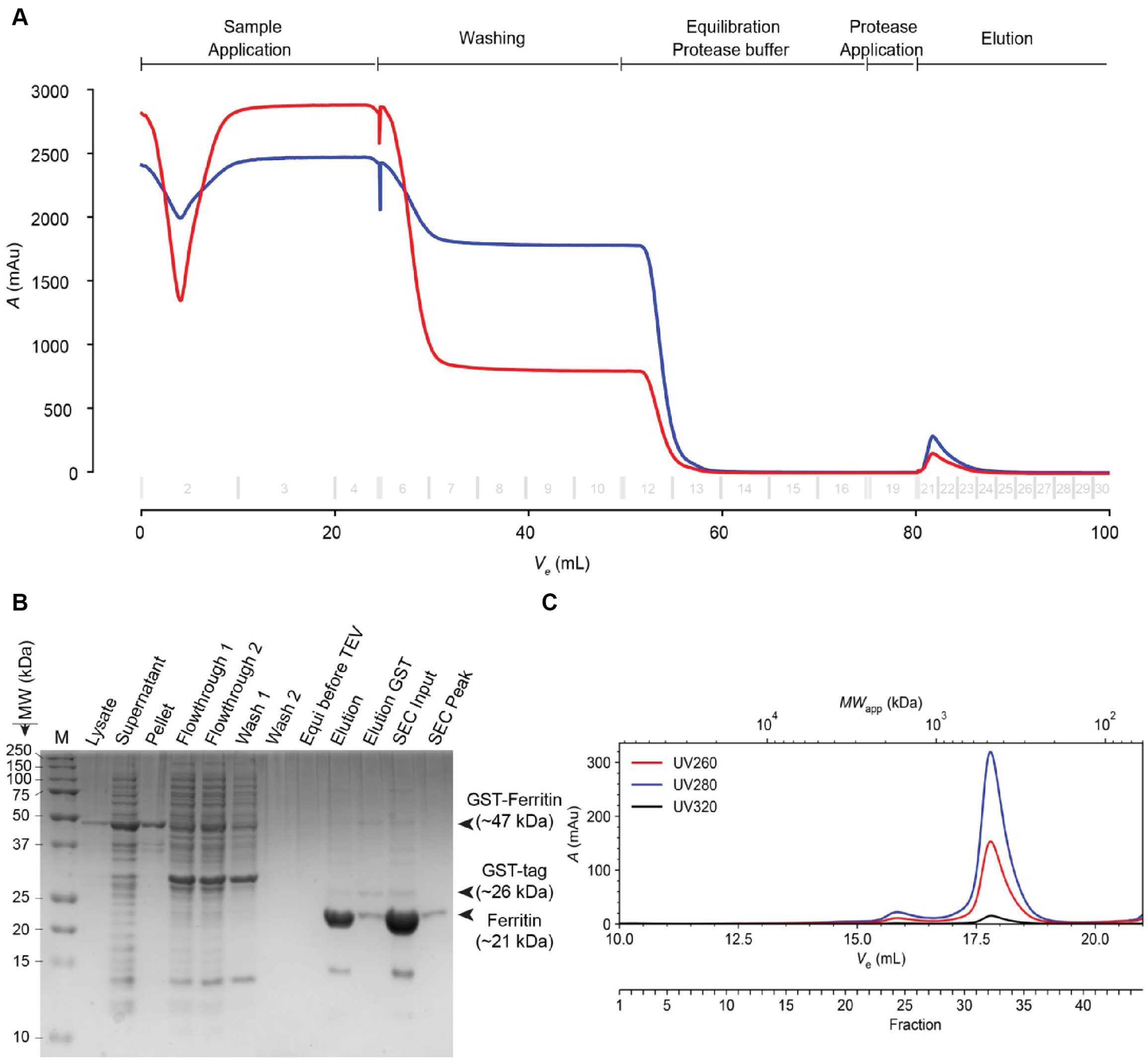

Fig. S1. Expression and Purification.

(A) Chromatogram of the purification of apoferritin from bacterial lysate. Purification phases are noted above the chromatogram and UV-absorption at 260 nm and 280 nm are drawn in red and blue respectively. The first peak after on-column digestion with TEV-protease was further purified using size-exclusion chromatography. (B) SDS-PAGE of the complete purification of apoferritin. GST-Ferritin, the GST-tag after TEV-cleavage and cleaved apoferritin are marked with arrows at their respective MW. C: SEC-Profile of cleaved apoferritin. UV-absorption at 260 nm, 280 nm and 320 nm are drawn in red, blue and black respectively and the apparent molecular weight is shown in a logarithmic scale above the chromatogram. The peak containing fully assembled apoferritin (Fractions 32 to 36, MW 400-500 kDa) was collected and subjected to pH and vitrification.

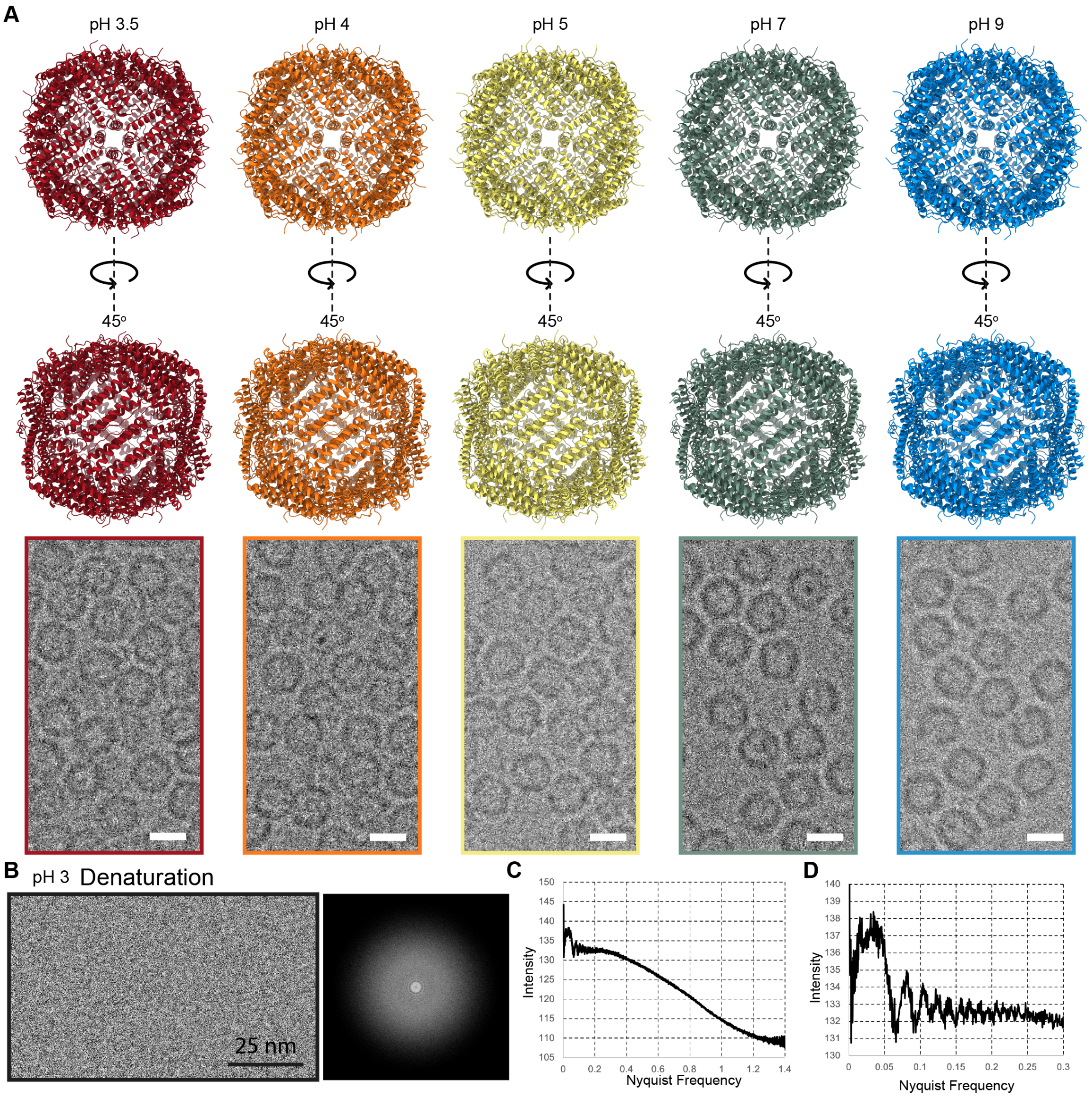

Fig. S2. Apoferritin models and representative micrographs across p*H* values.
(A) Resulting model and data collection representative micrograph for ApoF cryo-EM data collection at pH 3.5, pH 4, pH 5, pH 7 and pH 9. (B) At pH =< 3 there are no observable intact apoferritin particles visible on the cryo-EM micrographs, but the Fourier transform of the image shows protein presence. (C-D) Quantitative analysis of Fourier Transform (FT) intensities show oscillations at different Nyquist frequencies, indicating presence of proteinaceous material. Scale bars = 10 nm.

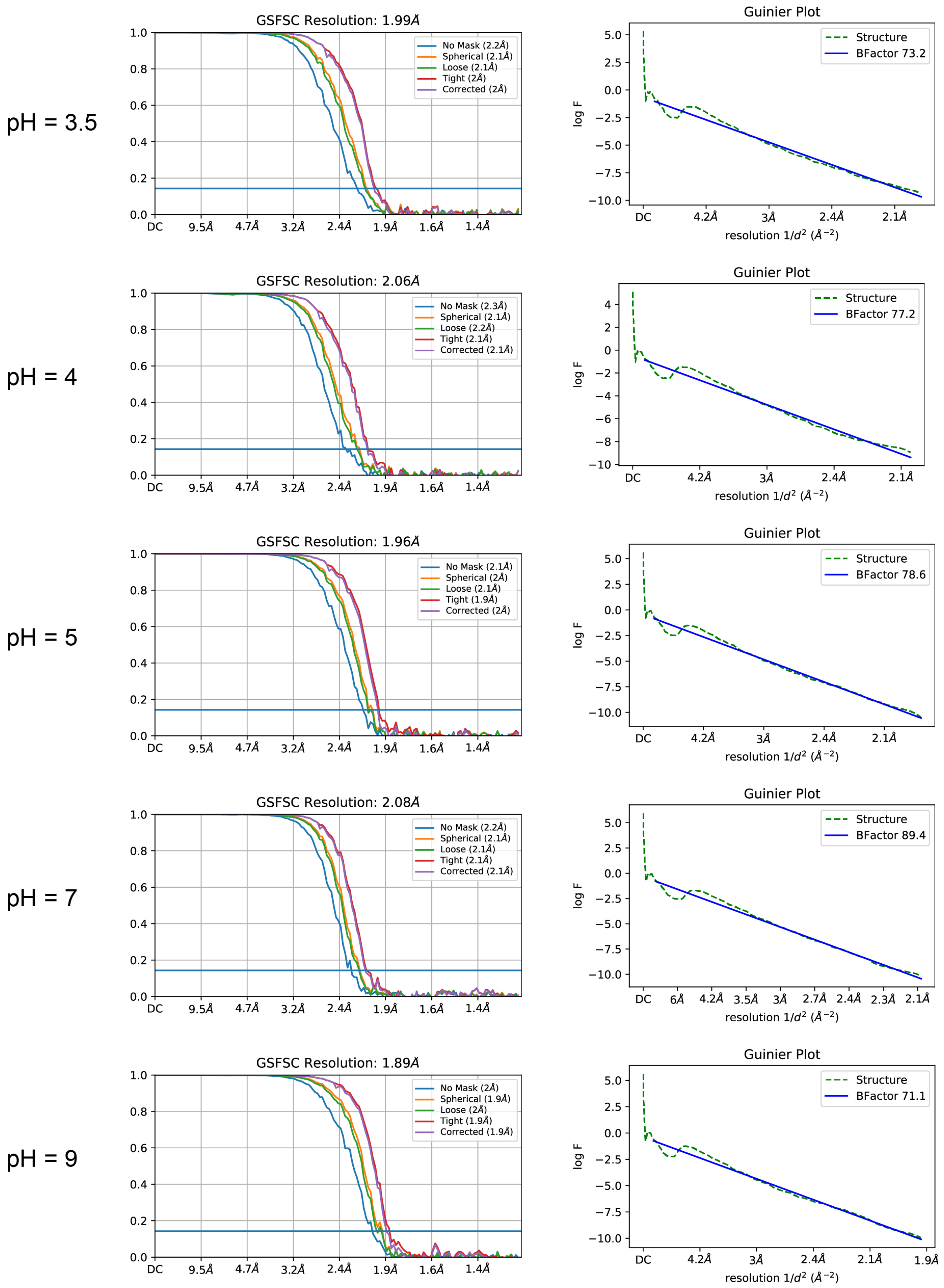

Fig. S3. Fourier Shell Correlation plots for each reconstruction.

FSC (Fourier Shell Correlation) and Guinier Plots for all reconstructions at the different p*H*s.

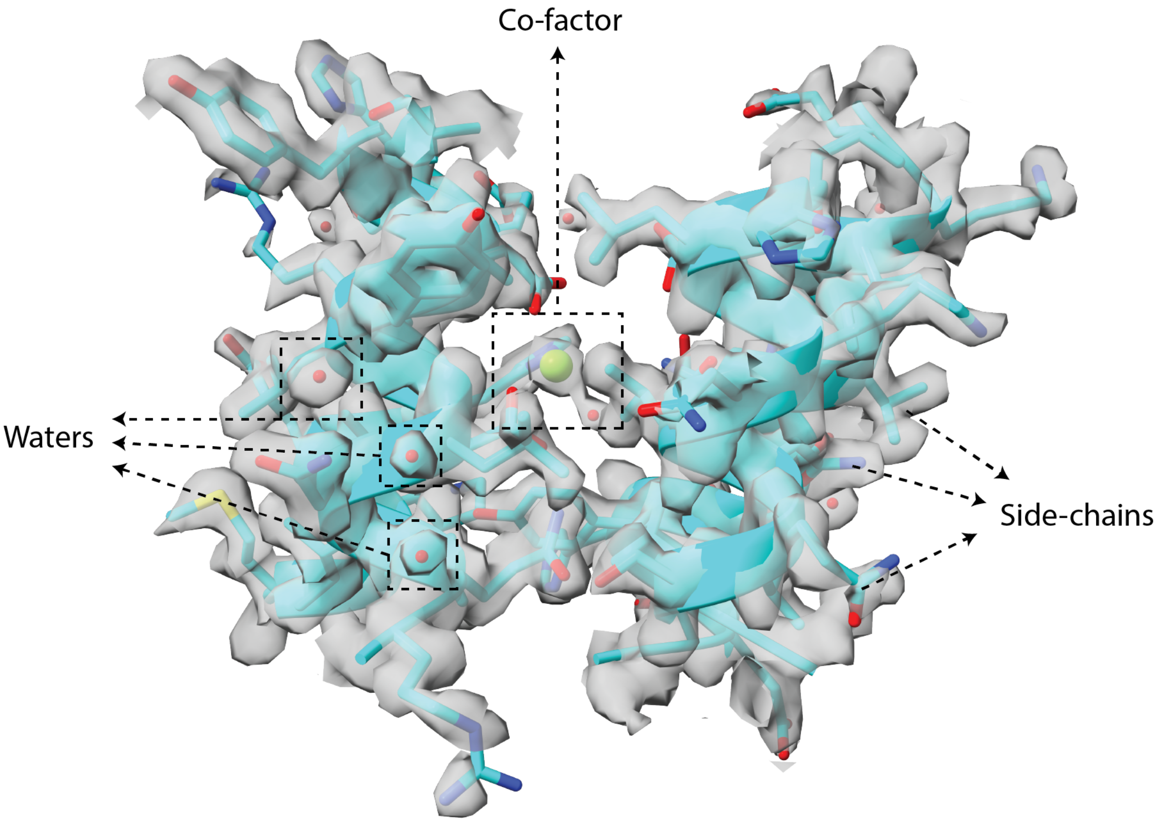

Fig. S4. Resolvability.

Example of resolvability for different molecule classifications in resolved cryo-EM maps. In all resolved structures, water molecules, co-factors (*e.g.,* magnesium) and side-chain densities were clearly visible and able to be modeled accordingly. Map threshold is 1.07, step 1.

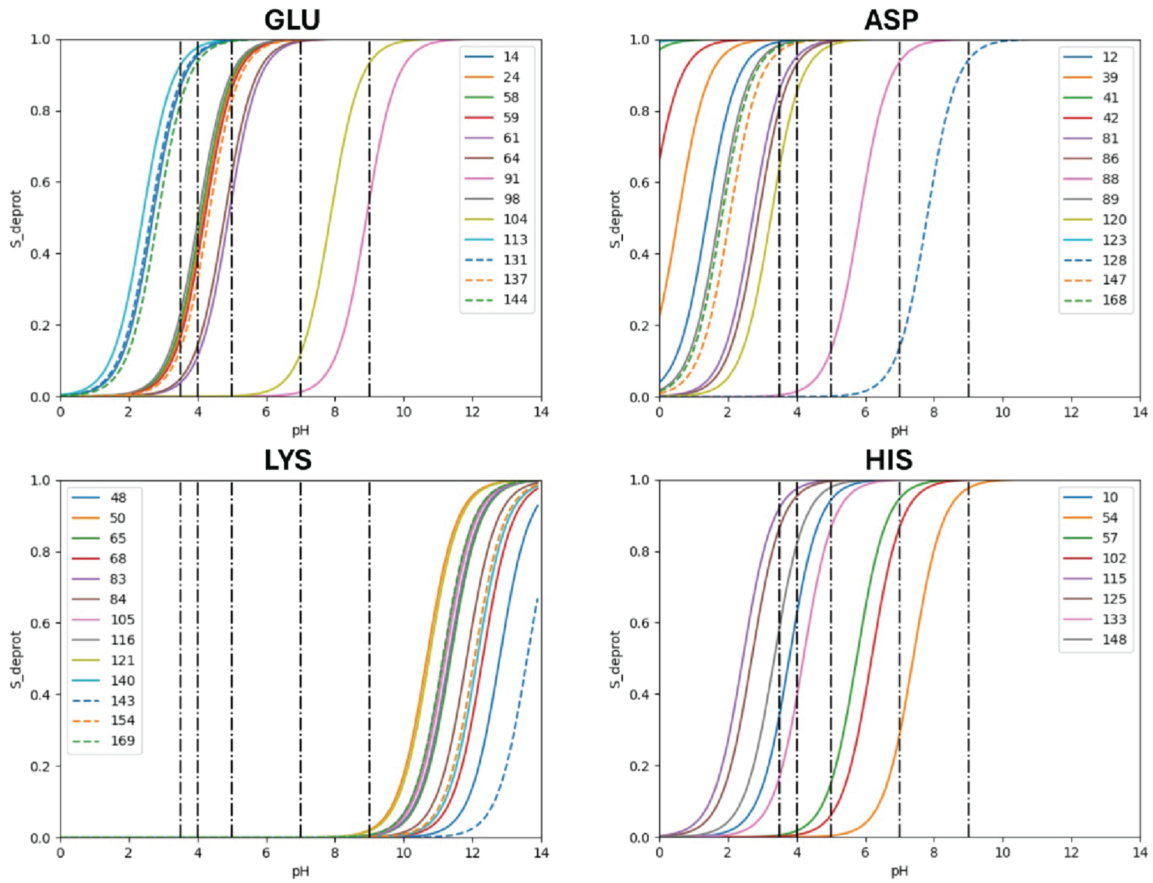

Fig. S5. Calculated titration curves for the side-chain functional groups of Asp, Glu, Lys and His residues in the apoferritin structure calculated by CpH MD simulations.

Vertical lines indicate the different pH values which should be adjusted in the whole-assembly apoferritin MD simulations.

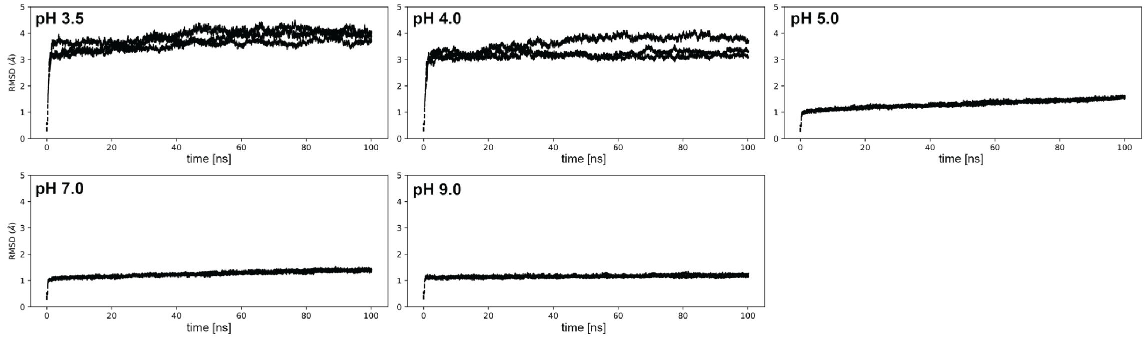

Fig. S6. RMSD-vs-time plots of the whole apoferritin complex relative to the cryo-EM structure in the MD simulations at different p*H* values.

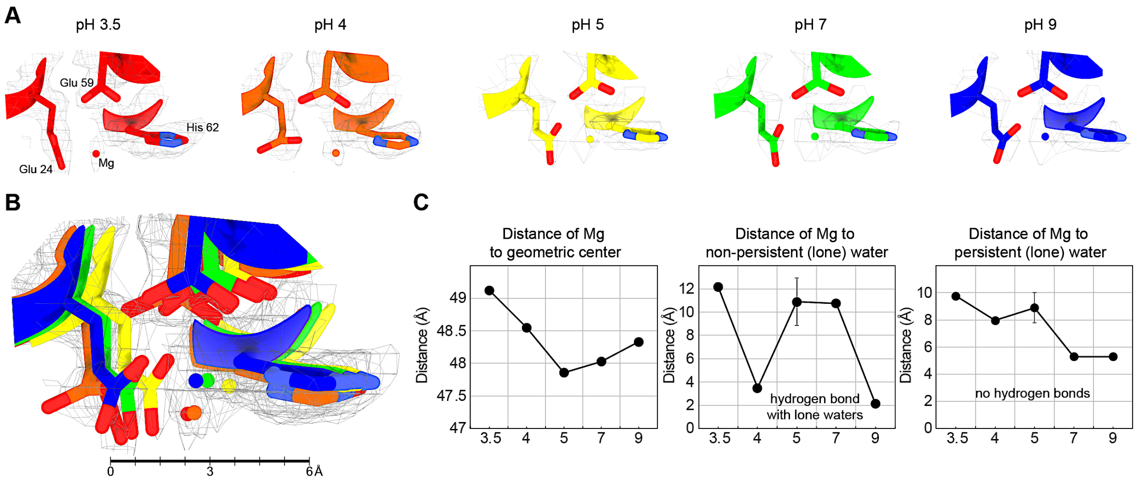

Fig. S7. Contacts of the magnesium ion.

(A) The magnesium ion remains stable as function of p*H*. **(B)** Local superposition of residues organizing the magnesium, and the 3D location of the ion, color-coded as function of p*H*. Its movement is minimal. **(C)** The magnesium shows a complex movement in respect to the geometric center (left plot), and hydrogen bonds only occur with "lone" water molecules, i.e., water molecules recovered in only a single p*H*. Right plot shows that Mg does not form hydrogen bonds with persistent water molecules as opposed to iron.

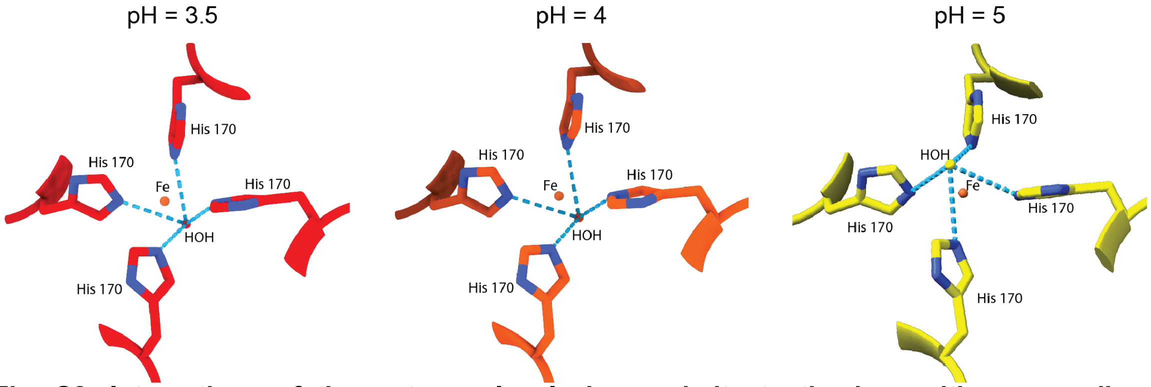

Fig. S8. Interactions of the water molecule in proximity to the iron with surrounding histidines.

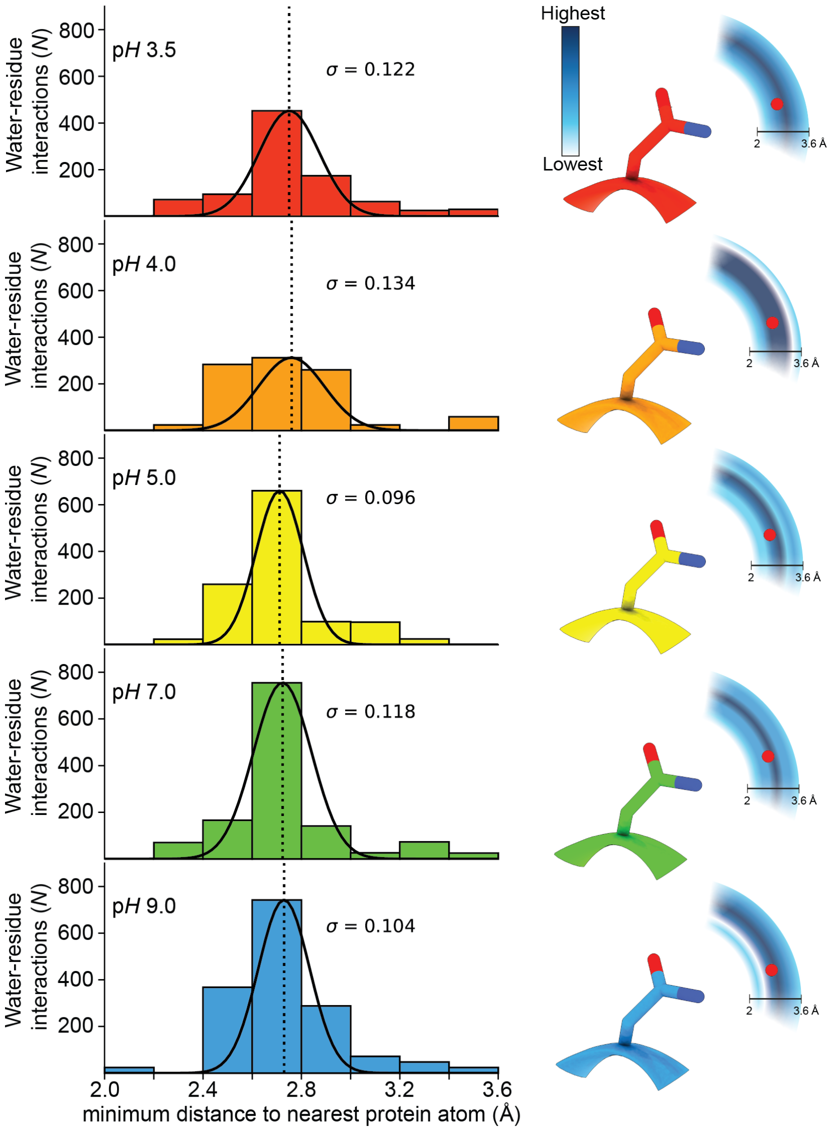

Fig. S9. Distributions of water-amino acid distances, considering the proximal amino acid. Distribution of distances of resolved water molecules to the nearest amino acid atom. There is a clear Gaussian distribution in all p*H*s with upper bounds at 3.6 Å, lower bounds at 2 Å and an average of ~2.7 Å. On the right, a 2D displacement diagram illustrates a water molecule adjacent to a protein residue, interpreting the distance distributions shown on the left.

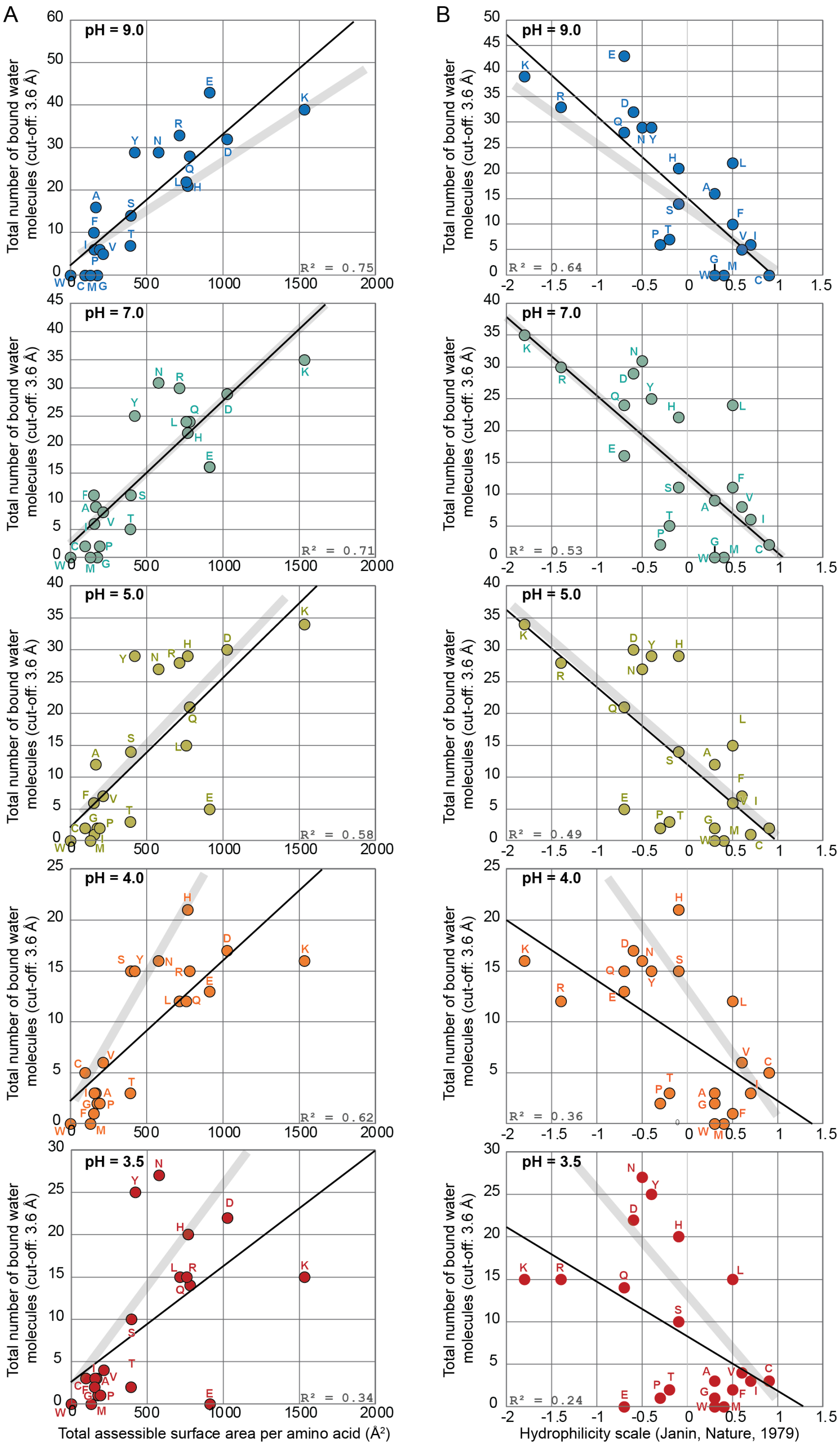

Fig. S10. Correlations of physical-chemical parameters with number of water molecules. Accessible surface area are strong predictors of protein solvation, but such descriptors are increasingly weaker with lower p*H*. Grey line represents the trend observed at p*H* 7. Note that values in x and y axes are aggregate numbers per amino acid.

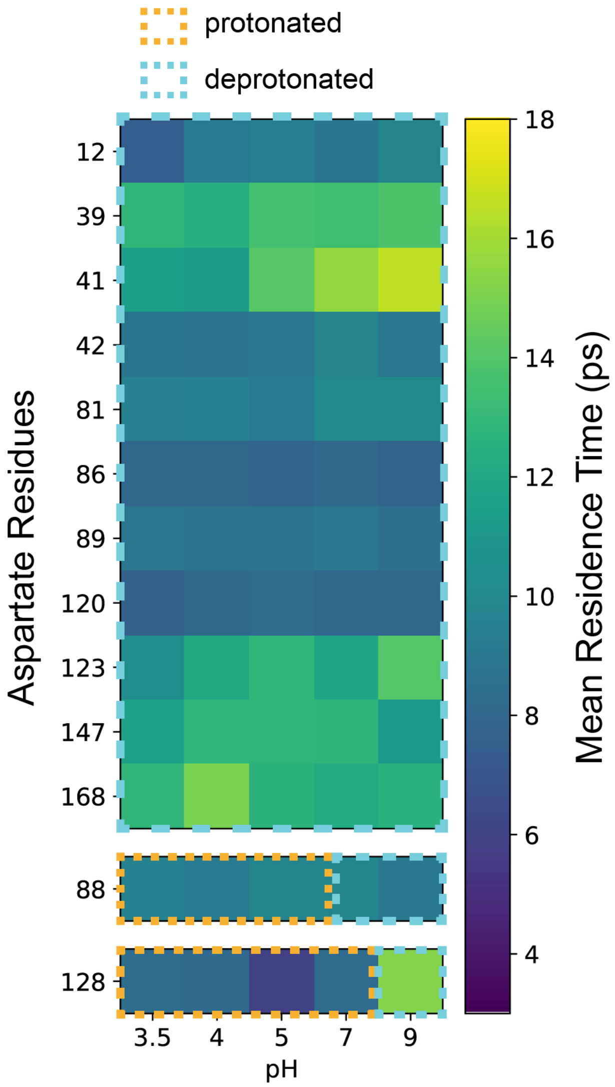

Fig. S11. Residence times of waters surrounding the aspartate residues at different p*H* values.

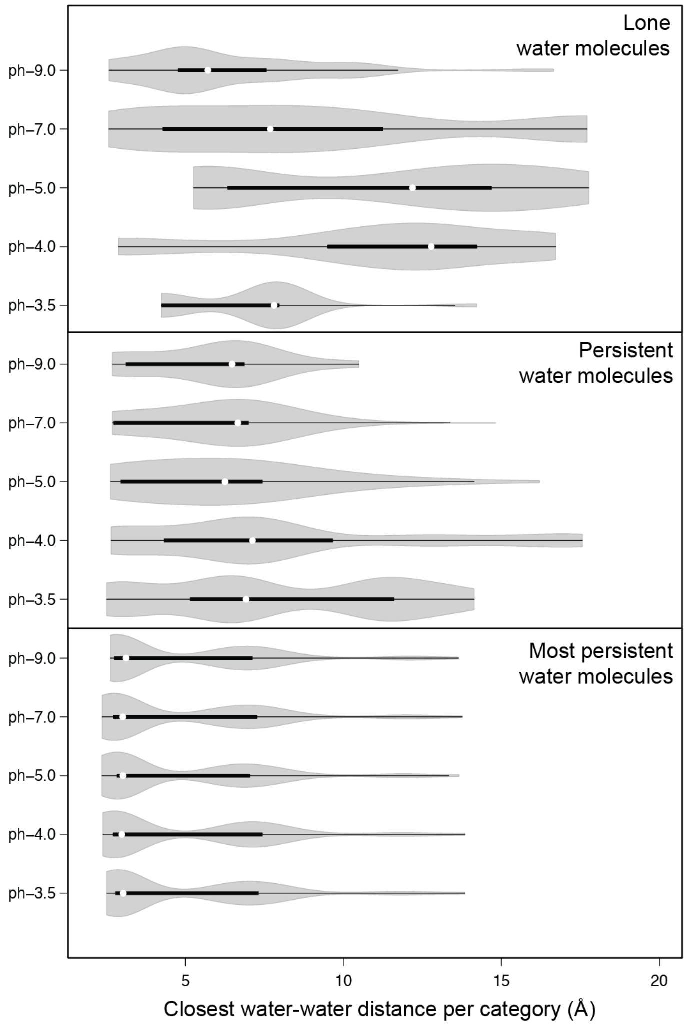

Fig. S12. Distribution of closest water-water distances for different categories of water molecules across p*H* conditions.

Violin plots show the distribution of minimum distances (Å) between water molecules classified as (top) lone water molecules, (middle) persistent water molecules, and (bottom) most persistent water molecules at p*H* 3.5, 4.0, 5.0, 7.0, and 9.0. White dots represent the median values, thick black bars indicate the interquartile range, and thin black lines show the 95% confidence intervals. The width of the gray violin plots reflects the density of observations at each distance value.

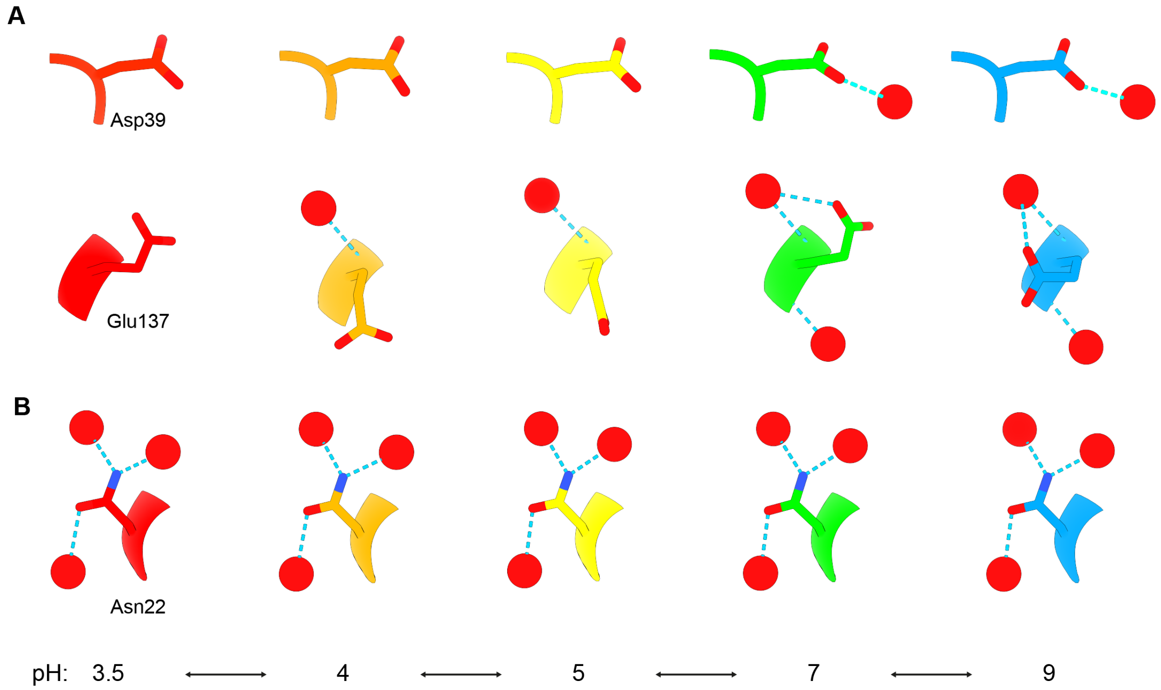

Fig. S13. p*H*-dependent water distribution. (A) Aspartate (Asp39) and glutamate (Glu137) residues show an increasing number of neighboring water molecules with rising p*H*. **(B)** In contrast, the asparagine residue (Asn22) maintains stably bound (persistent) water molecules across all p*H* values examined.

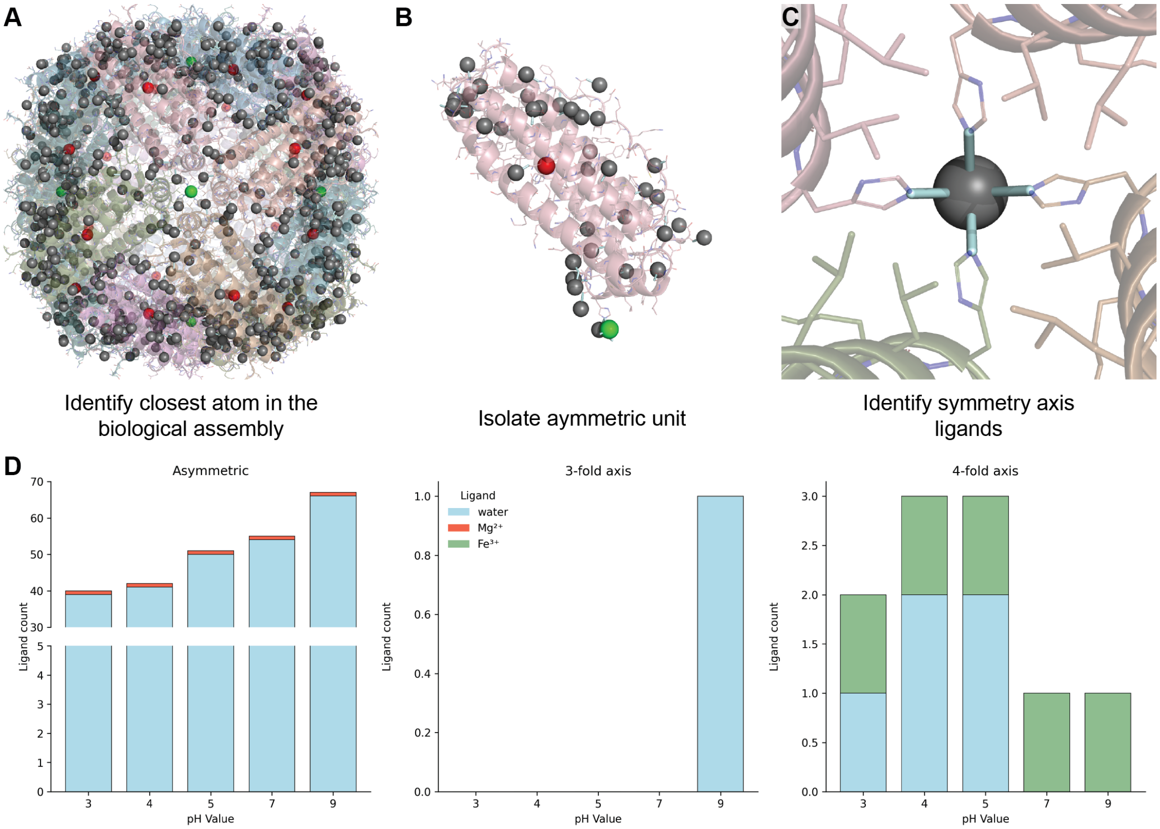

Fig. S14. Identification and classification of asymmetric and symmetry-related ligands in the apoferritin biological assembly.

(A) Cryo-EM reconstruction of the complete biological assembly refined in octahedral (O) symmetry, showing the protein (cartoon) and all validated solvent and ion sites (spheres). For each ligand, the closest protein atom across all chains of the assembly was identified based on Euclidean distance. **(B)** Isolation of a single asymmetric unit after ligand assignment. Following distance-based classification, all ligands were assigned to a chain, and only chain A was retained to represent the asymmetric unit. **(C)** Close-up example of a ligand located on a symmetry axis, illustrating equivalent coordinating residues from multiple chains used to identify symmetry-related sites. **(D)** Quantification of ligand types (water, Mg^{2+}, Fe^{3+}) across p*H* values, classified as asymmetric or symmetry-related (3-fold and 4-fold axes). Ligands were classified by comparing per-chain minimal ligand-atom distances within defined thresholds. Symmetry-related ligands were assigned occupancies according to the symmetry order (0.33 for 3-fold, 0.25 for 4-fold), while all ligands were ultimately mapped onto chain A to yield a fully annotated asymmetric unit.

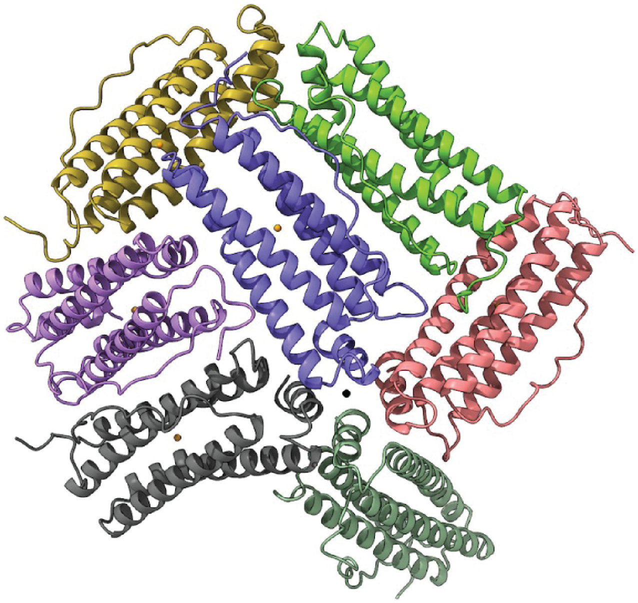

Fig. S15. Apoferritin 7-mer system for running CpH MD simulations.

The seven protein chains are displayed with different colors. A – dark purple, B - fuchsia, C - dark green, H - black, N - purple, P - yellow, Q – bright green. Magnesium and iron ions are colored orange and black, respectively. The titrated residues are listed in **Table S2**.

	#1 p*H* 3.5 (EMDB-54951) (PDB 9SJR)	#2 p*H* 4 (EMDB-54952) (PDB 9SJS)	#3 p*H* 5 (EMDB-54953) (PDB 9SJT)	#4 p*H* 7 (EMDB-54954) (PDB 9SJU)	#5 p*H* 9 (EMDB-54955) (PDB 9SJV)
**Data collection and processing**					
Magnification	240,000	240,000	240,000	240,000	240,000
Voltage (kV)	200	200	200	200	200
Image Nr.	3,725	2,308	2,541	1,891	1,867
Electron exposure (e–/Å^2)	30	30	30	30	30
Defocus range (μm)	1.0-2.0 μm	1.0-2.0 μm	1.0-2.0 μm	1.0-2.0 μm	1.0-2.0 μm
Pixel size (Å)	0.59	0.59	0.59	0.59	0.59
Symmetry imposed	O	O	O	O	O
Initial particle images (no.)	710,073	280,270	521,922	529,107	438,640
Final particle images (no.)	217,187	267,457	421,029	432,247	343,262
Map resolution (Å) FSC threshold	1.99 (0.143)	2.06 (0.143)	1.96 (0.143)	2.08 (0.143)	1.89 (0.143)
**Refinement**					
Map sharpening *B* factor (Å^2)	73.2	77.2	78.6	89.4	71.1
Model composition					
Non-hydrogen atoms	35016	35088	35304	35352	35664
Protein residues	4152	4152	4152	4152	4152
Ligands	48	48	48	48	48
Waters	960	1032	1248	1296	1608
B factors (Å^2)					
Protein	4.93/41.53/14.10	7.59/40.25/17.21	2.74/33.02/10.01	5.29/41.13/14.68	1.01/28.35/5.02
Ligand	5.08/24.64/14.86	8.38/27.77/18.07	8.06/13.43/10.75	12.17/20.10/16.14	2.08/4.77/3.42
Water	5.80/30.00/15.12	8.98/25.00/17.73	6.31/19.35/10.88	9.30/30.00/15.99	1.57/20.00/6.26
R.m.s. deviations					
Bond lengths (Å)	0.004	0.004	0.004	0.004	0.004
Bond angles (°)	0.904	0.886	0.889	0.938	0.898
Validation					
MolProbity score	1.36	1.45	1.70	1.55	1.32
Clashscore	5.12	8.26	7.37	7.03	3.67
Poor rotamers (%)	1.30	0.65	1.95	1.30	1.30
Ramachandran plot					
Favored (%)	98.83	98.25	97.66	97.66	97.66
Allowed (%)	1.17	1.75	2.34	2.34	2.34
Disallowed (%)	0.00	0.00	0.00	0.00	0.00

Table S1. Cryo-EM data collection, refinement and validation statistics.

p*H*	Comparison	KS statistic	*p-value*	$Mean_1$	$Mean_2$	Med_1	Med_2	N_1	N_2	SD_1	SD_2
all	always vs persistent	0.387	5.0×10^{-44}	5.00	6.44	2.95	6.58	499	940	2.80	2.51
all	always vs lone	0.439	1.7×10^{-54}	5.00	8.48	2.95	7.68	499	822	2.80	4.45
all	persistent vs lone	0.294	8.1×10^{-34}	6.44	8.48	6.58	7.68	940	822	2.51	4.45
3.5	always vs persistent	0.365	1.2×10^{-25}	5.05	7.97	3.03	6.92	499	368	2.78	3.78
3.5	always vs lone	0.561	1.1×10^{-19}	5.05	6.97	3.03	7.80	499	75	2.78	2.25
3.5	persistent vs lone	0.389	5.5×10^{-9}	7.97	6.97	6.92	7.80	368	75	3.78	2.25
4.0	always vs persistent	0.354	3.0×10^{-22}	5.05	8.00	2.98	7.12	499	320	2.86	4.62
4.0	always vs lone	0.732	2.6×10^{-69}	5.05	11.14	2.98	12.78	499	177	2.86	4.31
4.0	persistent vs lone	0.534	2.3×10^{-30}	8.00	11.14	7.12	12.78	320	177	4.62	4.31
5.0	always vs persistent	0.245	4.6×10^{-15}	4.98	6.04	3.02	6.25	499	615	2.76	2.91
5.0	always vs lone	0.569	3.1×10^{-25}	4.98	11.34	3.02	12.19	499	98	2.76	4.74
5.0	persistent vs lone	0.553	1.4×10^{-24}	6.04	11.34	6.25	12.19	615	98	2.91	4.74
7.0	always vs persistent	0.309	5.0×10^{-24}	4.95	5.86	3.01	6.66	499	634	2.83	2.16
7.0	always vs lone	0.500	2.1×10^{-25}	4.95	9.13	3.01	7.68	499	139	2.83	5.18
7.0	persistent vs lone	0.484	6.1×10^{-25}	5.86	9.13	6.66	7.68	634	139	2.16	5.18
9.0	always vs persistent	0.319	1.1×10^{-27}	5.00	5.81	3.12	6.47	499	760	2.77	2.13
9.0	always vs lone	0.381	2.6×10^{-26}	5.00	6.28	3.12	5.71	499	333	2.77	2.92
9.0	persistent vs lone	0.287	2.6×10^{-17}	5.81	6.28	6.47	5.71	760	333	2.13	2.92

Table S2. Kolmogorov-Smirnov (KS) statistics and summary of water–protein distance distributions.

Pairwise comparisons were performed between always present, persistent (sometimes present), and lone (single-site) water molecules across all p*H* values and within each p*H* condition (3.5, 4.0, 5.0, 7.0, 9.0). For each comparison, the KS statistic and *p-value* are shown, as well as mean and median distances for both groups ($Mean_1$/$Mean_2$, $Median_1$/$Median_2$), sample sizes (N_1/N_2), and standard deviations (SD_1/SD_2). Global values (p*H* all) correspond to the aggregate distributions of all waters across all conditions.

Apoferritin protomer	Titrated residues
A	all except for E24, E59, H62 (proton on NE2), H170 (proton on ND1)
B	K143, D147, K154
C	-
H	D39, D41, D42, K46, K169
N	H115, D123, D128, E131
P	H115, D128, E131, K143
Q	D39, H57, E64, K68, D81, K83, K84

Table S3. Overview of titrated residues in the apoferritin protomers. Residues listed were adjusted during constant-p*H* simulations.

Protonation states are specified where relevant (e.g., H62 protonated on NE2, H170 protonated on ND1).

Simulation	Box size (Å*Å*Å)	Protein atoms / waters / ions / Buffer	Replicates	Time / replicate	Total time
CpH MD, p*H* 4	142 * 142 * 99	19575 / 59236 / 24 / 141	1 (at 25 p*H* values)	100 ns *25	2500 ns
CpH MD, p*H* 7	142 * 142 * 99	19575 / 58651 / 24 / 141	1 (at 25 p*H* values)	100 ns *25	2500 ns
CpH MD, p*H* 9	140 * 140 * 97	19575 / 56514 / 24 / 141	1 (at 25 p*H* values)	100 ns *25	2500 ns
Classic MD, p*H* 3.5	155 * 155 * 155	67205 / 103355 / 988 / None	3	100 ns	300 ns
Classic MD, p*H* 4	153 * 153 * 153	67181 / 99370 / 938 / None	3	100 ns	300 ns
Classic MD, p*H* 5	151 * 151 * 151	66989 / 95194 / 720 / None	3	100 ns	300 ns
Classic MD, p*H* 7	151 * 151 * 151	66917 / 95303 / 724 / None	3	100 ns	300 ns
Classic MD, p*H* 9	155 * 155 * 155	66821 / 104149 / 870 / None	3	100 ns	300 ns

Table S4. Summary of molecular dynamics simulation setups.

For each simulation condition, the table reports the simulation type (constant-p*H* or classical MD), target p*H*, box dimensions, and system composition, including the number of protein atoms, water molecules, ions (Na^+/Cl^-), and buffer components where applicable. The number of independent replicas, simulation length per replica, and total accumulated simulation time are also indicated. Constant-p*H* MD simulations were performed with a single replica spanning 25 discrete p*H* values, whereas classical MD simulations were carried out with three independent replicas per p*H*. This table is provided to explicitly document system size, solvation, and ionic content, enabling direct comparison of simulation conditions across p*H* values.
